# Scaling analysis for the investigation of slip mechanisms in nanofluids

**DOI:** 10.1186/1556-276X-6-471

**Published:** 2011-07-26

**Authors:** S Savithiri, Arvind Pattamatta, Sarit K Das

**Affiliations:** 1 Heat Transfer and Thermal Power Laboratory, Department of Mechanical Engineering, Indian Institute of Technology - Madras, Chennai 600 036, India

## Abstract

The primary objective of this study is to investigate the effect of slip mechanisms in nanofluids through scaling analysis. The role of nanoparticle slip mechanisms in both water- and ethylene glycol-based nanofluids is analyzed by considering shape, size, concentration, and temperature of the nanoparticles. From the scaling analysis, it is found that all of the slip mechanisms are dominant in particles of cylindrical shape as compared to that of spherical and sheet particles. The magnitudes of slip mechanisms are found to be higher for particles of size between 10 and 80 nm. The Brownian force is found to dominate in smaller particles below 10 nm and also at smaller volume fraction. However, the drag force is found to dominate in smaller particles below 10 nm and at higher volume fraction. The effect of thermophoresis and Magnus forces is found to increase with the particle size and concentration. In terms of time scales, the Brownian and gravity forces act considerably over a longer duration than the other forces. For copper-water-based nanofluid, the effective contribution of slip mechanisms leads to a heat transfer augmentation which is approximately 36% over that of the base fluid. The drag and gravity forces tend to reduce the Nusselt number of the nanofluid while the other forces tend to enhance it.

## Introduction

Nanofluid was first proposed by Choi and Eastman [1] about a decade ago, to indicate engineered colloids composed of nanoparticles dispersed in a base fluid. Contrary to the milli- and micro-sized particle slumped explored in the past, nanoparticles are relatively close in size to the molecules of the base fluid and thus can realize very stable suspensions with little gravitational settling over long periods of time. It has long been recognized that suspensions of solid particles in liquid have great potential as improved heat management fluids. The enhancement of thermal transport properties of nanofluids was even greater than that of suspensions of coarse-grained materials. In the recent years, many studies show that there is an abnormal increase in single phase convective heat transfer coefficient relative to the base fluid [2]. Such an increase mainly depends on factors such as the form and size of the particles and their concentration, the thermal properties of the base fluid as well as those of the particles, kinetics of particle in flowing suspension, and nanoparticle slip mechanisms. The enhancement mechanism of heat transfer in nanofluid can be explained based on the following two aspects: (1) The suspended nanoparticles increase the thermal conductivity of the two-phase mixture and (2) the chaotic movement of the ultrafine particles due to the slip between the particles and the base fluid resulting in thermal dispersion plays an important role in heat transfer enhancement. Slip mechanisms of the particles increase the energy exchange rates in the nanofluid. Thermal dispersion will flatten the temperature distribution inside the nanofluid and make the temperature gradient between the fluid and wall steeper, which augments heat transfer rate between the fluid and the wall [3]. Understanding the effect of different forces that bring about the slip mechanism is therefore essential in the study of convective transport of nanofluids.

An overall understanding of the effect of nanoparticle slip mechanisms for the augmentation of heat transport in nanofluids is in its infancy. In the past, several authors have attempted scaling analysis for convective transport of nanofluids to show the effect of slip mechanisms. Scaling analysis [4-6] is an effective tool to apply and develop mathematical models for describing transport processes. Through scaling analysis, the solution for any quantity that can be obtained from the governing equations can be reduced to a function of the dimensionless independent variables and the dimensionless groups. Ahuja [7] examined the augmentation in heat transport of flowing suspensions due to the contribution of rotational and translational motions by an order of magnitude analysis and concluded that the translational motion is expected to be negligibly small compared to that of the rotational motion of the particles. Savino and Paterna [8] performed order of magnitude analysis for Soret effect in water/alumina nanofluid and concluded that the thermofluid-dynamic behavior may be influenced by gravity and the relative orientation between the residual gravity vector and the imposed temperature gradient. Khandekar et al. [9] used scaling analysis for different nanofluids to show that entrapment of nanoparticles in the grooves of surface roughness leads to deterioration of the thermal performance of nanofluid in closed two-phase thermosyphon. Hwang et al. [10], in his study for water/alumina nanofluid, showed that both thermophoresis and Brownian diffusion have major effect on the particle migration and that the effect of viscosity gradient and non-uniform shear rate can be negligible. Buongiorno [11] estimated the relative importance of different nanoparticle transport mechanisms through scaling analysis for water/alumina nanofluid and concluded that Brownian diffusion and thermophoresis are the two most important slip mechanisms. Also, he ascertained that these results hold good for any nanoparticle size and nanofluid combination.

However, the different slip mechanisms between nanoparticle and the base fluid are dependent on several factors such as the shape, size, and volume fraction of the particle. Also, the thermophysical properties of the nanofluid used in the scaling analysis affect the magnitude of the slip forces in nanofluids which were not taken into consideration in the previous studies discussed above. Therefore, the objective of the present work is to carry out a detailed scaling analysis to understand the effect of seven different slip mechanisms in both water- and ethylene glycol-based nanofluids. A comprehensive parametric study has been carried out by varying the shape, size, concentration, and temperature of the nanoparticle in the fluid in order to understand the relative effect of these parameters on the magnitude of slip forces. The study is extended across different nanoparticles such as gold, copper, alumina, titania, silica, carbon nanotube (CNT), and graphene, suspended in the base fluid. The effect of slip mechanism on heat transfer augmentation in these nanofluids due to the slip mechanisms is also studied.

### Governing equations

The fluid surrounding the nanoparticles will be assumed to be continuum. Knudsen number is defined as the ratio of the molecule mean free path of base fluid molecules to the nanoparticles diameter [11]:(1)

where *d*_p _is the particle diameter and *λ *is the molecule mean free path of base fluid molecules and is given by:(2)

where *R *is the universal gas constant, *T *is the temperature, *d*_m _is the molecular diameters of base fluid, *N*_A _is the Avagodra's constant, and *P *is the pressure.

For water and ethylene glycol, the values of molecular mean free path are 0.278 and 0.26 nm, respectively. Therefore, for the nanoparticles in range of interest (1-100 nm), the Knudsen number is relatively small (Kn < 0.3); thus, the assumption of continuum is reasonable.

### Continuous fluid phase

The governing equations for the continuous phase include the continuity equation (mass balance), equation of motion (momentum balance), and energy equation (energy balance). They are given, respectively, in the following:

Continuity equation(3)

Momentum equation(4)

Energy equation(5)

*T*_bf _in Equation 2 is the stress tensor defined as:(6)

where *μ*_bf _is the shear viscosity of the base fluid phase and *I *is the unit vector. *S*_p _in Equation 4 is the source term representing the momentum transfer between the fluid and particle phases and is obtained by computing the momentum variation due to several forces of slip, ∑ *F*, experienced by the control volume as:(7)

### Nanoparticle

In the Lagrangian frame of reference, the equation of motion of a nanoparticle is given by:(8)

The equation of motion of nanoparticles contains the drag force *F*_D_, gravity *F*_G_, Brownian motion force *F*_B_, thermophoresis force *F*_T_, Saffman's lift force *F*_L_, rotational force *F*_R_, and Magnus effect *F*_M _and is given in the following equation:(9)

The abbreviations for the different forces are listed in Table 1.

**Table 1:** Abbreviations for different forces

Abbreviations	*F*_D_	*F*_G_	*F*_B_	*F*_T_	*F*_L_	*F*_R_	*F*_M_
Forces	Drag	Gravity	Brownian	Thermophoresis	Lift	Rotational	Magnus effect

The coupling between the continuous fluid phase and discrete phase is realized through the Newton's third law of motion. The inter-particle forces such as the Van der Waals and electrostatic forces are neglected in the analysis due to their relatively negligible contributions in nanofluids.

The above forces are computed separately as shown below.

#### Drag force

Drag is the force generated in opposition to the direction of motion of a particle in a fluid. Drag force is proportional to the relative velocity between the base fluid and nanoparticle and is expressed by [12]:(10)

where *v*_bf _is the velocity of the base fluid, *v*_p _is the particle velocity, *m*_p _is the mass of the particle, *β *is the interphase momentum exchange coefficient:(11)

*Re*_D _is the Reynolds number due to drag:(12)

*C*_D _is the drag coefficient for spherical particles and is given by:(13)

For non-spherical particles [13]:(14)

where:(14a)(14b)(14c)(14d)

Here, Ψ is the shape factor:(14e)

where *A*_sp _is the surface area of the sphere of the same volume as the non-spherical particle:(14f)

and *A *is the actual surface area of the non-spherical particle.

#### Gravity

Gravity force is proportional to the volume of the particle, and the relative density of nanoparticle and base fluid is expressed as:(15)

where *V*_p _is the volume of the particle, *ρ*_p _is the density of the nanoparticle, *ρ*_bf _is the density of the base fluid, and *g *is acceleration due to gravity.

#### Brownian force

The random motion of nanoparticles within the base fluid is called Brownian motion and results from continuous collisions between the nanoparticles and the molecules of the base fluid. Brownian force is a function of concentration gradient, surface area of the particle, and the Brownian diffusion coefficient [11]:(16)

where *D*_B _is the Brownian diffusion coefficient (*D*_B_) for spherical particles [11]:(16a)

For non-spherical particles:(16b)

where *K*_B _is the Boltzmann constant, *T *is the temperature, *h *is the length of the non-spherical particle, *d*_p _is the particle diameter, *μ*_nf _is the dynamic viscosity of the nanofluid. and *v*_B _is the Brownian velocity and is a function of temperature and diameter of the particle.

For spherical particles:(16c)

For non-spherical particles:(16d)

#### Thermophoresis force

Small particles suspended in a fluid that has a temperature gradient experience a force in the direction opposite to that of the gradient. This phenomenon is known as the thermophoresis. Thermophoresis is a function of thermophoretic velocity, temperature gradient, Knudsen number, thermal conductivity, dynamic viscosity, and density of the nanofluid [11]:(17)

where *ϕ *is the volume fraction of the particle, *v*_T _is a thermophoretic velocity:(18)

where *ρ*_nf _is the density of nanofluid, *μ*_nf _is the dynamic viscosity of the nanofluid, ∇*T *is the temperature gradient, and Kn is the Knudsen number:

*k*_nf _is the thermal conductivity of nanofluid and *k*_p _is the particle thermal conductivity.

#### Saffman's lift force

A free-rotating particle moving in a shear flow gives rise to a lift force. Lift due to shear, *F*_L _has been derived by Saffman [14] and it can be expressed as [15]:(19)

where *r *is the radius of the particle, *v*_bf _is the velocity of the base fluid, *v*_nf _is the kinematic viscosity of the fluid,  is the shear rate , *K*_L _= 81.2, and *D *is the diameter of the tube.

#### Particle rotational force

The force experienced by the particle due to rotational motion around a fixed axis is given as [7]:(20)

#### Magnus force

Under the effect of the shear stress, a particle rotates about an axis perpendicular to the main flow direction. If a relative axial velocity exists between the particle and the fluid, a force perpendicular to the main flow direction will arise. This is known as the Magnus effect. It is a function of the difference between the axial velocity and radial velocity of the particle [11]:(21)

The empirical relation proposed by Segre and Silberberg [16] for the velocity of radial motion of the particles (*v*_M_) is used:(22)

where  is the mass flow rate of the particle, *v*_m _is the mean velocity, , and .

### Thermophysical properties of nanofluids

The correlations used to compute the physical and thermal properties of the nanofluids are listed in Table 2. In this table, the subscripts p, bf, and nf refer to the particles, the base fluid, and the nanofluid, respectively. The density and specific heat of nanofluid are assumed to be a linear function of volume fraction due to lack of experimental data on their temperature dependence. Widely accepted correlations for determining the dynamic viscosity and thermal conductivity as a function of volume fraction for different nanofluids as shown in Table 2 are used in the analysis. For Al_2_O_3_-water nanofluids, the equation for effective thermal conductivity as suggested by Li and Peterson [17] is used.

**Table 2:** Thermophysical properties of nanofluids

Properties	Correlations	Nanofluids	Ref
Density	*ρ*_nf _= *ϕρ*_p _+ (1 - *ϕ*)*ρ*_bf_		**-**
Specific heat	(*ρC*_nf_) = *ϕ*(*ρC*_p_)_p _+ (1 - *ϕ*) (*ρC*_p_)_bf_		[21]
Dynamic viscosity	*μ*_nf _= *μ*_bf _(*T*) (1 + 2.5*ϕ*)	Water/Cu, gold, CNT, grapheme	[22]
	*μ*_nf _= *μ*_bf _(*T*) (1 + 39.11*ϕ *+ 533.9*ϕ*^2^)	Water/alumina	[11]
	*μ*_nf _= *μ*_bf _(*T*) (1 + 5.45*ϕ *+ 108.2*ϕ*^2^)	Water/titania	[11]
	*μ*_nf _= *μ*_bf _(*T*) (1 + 56.5*ϕ*)	Water/silica	[23]
	*μ*_nf _= *μ*_bf _(*T*) (1 + 11*ϕ*)	EG/Cu, gold, CNT, grapheme	[24]
	*μ*_nf _= *μ*_bf _(*T*) (1 - 0.19*ϕ *+ 3.6*ϕ*^2^)	EG/alumina	[25]
	*μ*_nf _= *μ*_bf _(*T*) (1 + 10.6*ϕ *+ 112.36*ϕ*^2^)	EG/titania	[26]
		EG/silica	[27]
Thermal conductivity	*k*_nf _- *k*_bf _= *k*_bf _(0.764*ϕ *+ 0.0186*T *- 0.46215)	Water/alumina, EG/alumina	[17]
	*k*_nf _= *k*_bf _(1 + 2.92*ϕ *- 11.99*ϕ*^2^)	Water/titania, EG/titania	[11]
		Other nanofluids	Maxwell's model

### Scaling analysis methodology

In this section, the forces contributing to the slip between the particle and base fluid are analyzed through scaling analysis. A Reynolds number is introduced for each force depending on the velocity of the particle and the base fluid velocity. The time scale is defined as the time that a nanoparticle takes to diffuse a length scale that is equal to its diameter under the effect of that mechanism. In the present study, scaling analysis is to understand the order of magnitude for the forces involved in the slip mechanism of nanofluids.

#### Drag

Mass of the particle:(23)

The drag force acting on the particle, *F*_D _is given by:(24)

The time scale for drag:(25)

#### Gravity

The velocity of nanoparticle due to gravitational settling, *v*_G_, can be calculated from a balance of buoyancy and viscous forces[11]:(26)(26a)

The corresponding Reynolds number and the force due to gravity can be expressed as:(27)

and the time scale for gravity:(28)

#### Brownian force

The corresponding Reynolds number and the force due to Brownian motion can be expressed as:(29)

and the time scale for Brownian:(30)

#### Thermophoresis

The corresponding Reynolds number and the force due to thermophoresis can be expressed as:(31)

and the time scale for thermophoresis:(32)

#### Saffman's lift force

The corresponding Reynolds number and the force due to lift can be expressed as:(33)

and the time scale for lift:(34)

#### Rotational force

The corresponding Reynolds number and the force due to particle rotation can be expressed as:(35)

and the time scale for rotational:(36)

#### Magnus effect

The corresponding Reynolds number and the force due to Magnus effect can be expressed as:(37)

and the time scale for Magnus effect:(38)

Applying the scaling analysis, to Equation 8, the acceleration term is normalized by the ratio of particle velocity to the relaxation time of the particle:(39)

The particle relaxation time can be expressed as [11]:(40)

and the particle Reynolds number as:(41)

By substituting all the forces in Equation 9, we get the Reynolds number of the particle as a function of the Reynolds numbers of all the seven slip mechanisms:(42)

Thus, from scaling analysis, we arrive at an expression for the particle Reynolds number which is dependent on the Reynolds number of each of the slip mechanisms, the relaxation time of the particle, and the time scale of each mechanism.

## Results and discussion

For the present study, nanoparticle suspensions flowing through a horizontal circular tube is considered as the model system. All the forces are calculated based on the equations derived in Section 4. The temperature-dependent nanofluid properties listed in Table 2 are used for calculating the forces described in Section 4. As an initial guess, the relative velocity between the fluid and particle is obtained by assuming Poiseuille flow [15]:(43)

Since the effect of forces on the velocity of particle cannot be incorporated in the above correlation, an iterative procedure is used to calculate the final velocity of the particle from the particle force balance, given by Equation 8. Based on the assumptions that the particle-particle interactions are negligible and that the density of a nanoparticle is greater than the density of the base fluid, the Equation 8 can be expressed as [18]:(44)

The above equation is iteratively solved by using the initial guess for *v*_p _from Equation 43 until the velocity of the particle is stabilized. A detailed parametric study is conducted to study the relative importance of different slip mechanisms by varying parameters such as shape, size, concentration, and temperature of the particle in the nanofluid as shown in Table 3. These mechanisms are analyzed for both water and ethylene glycol based nanofluids separately. The parametric study is done to study the shape effect for three different shapes, namely spherical, cylindrical, and sheet, while fixing the particle size to be 100 nm, volume fraction as 1%, and temperature as 20°C. The size effect is studied by varying the particle diameter between 1 and 80 nm, while fixing the shape of the particle to be cylindrical and considering the volume fraction and temperature to be 1% and 20°C, respectively. For the study involving variation of particle concentration, the concentration is varied between 0.5% to 5%, while the size of the nanoparticle is 10 nm and at a temperature of 20°C. The parametric study on temperature is conducted by fixing the volume fraction of nanoparticles to be 1% and the temperature between 20°C and 70°C. The effect of each of the parameters on the slip mechanisms are explained in the following sections.

**Table 3:** Parameters and their ranges used for scaling analysis

Parameters	Particle shape	Particle size (nm)	Particle concentration (%)	Particle temperature (°C)
Parametric study				
Particle shape	Spherical	100	1	20
	Cylindrical	80	1	20
	Sheet	80	1	20
Particle size	Cylindrical	1-80	1	20
Particle concentration	Cylindrical	10	0.5-5	20
Particle temperature	Cylindrical	10	1	20-70

### Effect of particle shape

**Figure 1:**
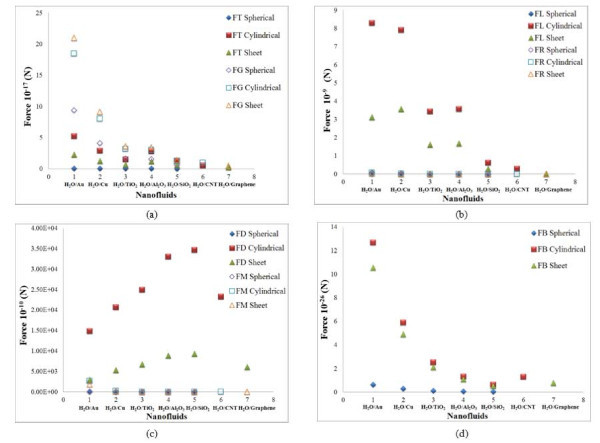
**Effect of particle shape**. (a) Gravity and thermophoresis forces, (b) lift and rotational forces, (c) drag and Magnus forces, and (d) Brownian force for water-based nanofluids.

The result of the scaling analysis on the particle shape is plotted in Figure 1. The results are plotted for water based nanofluids. From this figure, it is observed that all the seven slip mechanisms, namely the drag, rotational, gravity, thermophoresis, Brownian, Saffman lift, and Magnus forces, dominate for cylindrical particles compared to that of sheet and spherical particles. In terms of the order of magnitude, the drag force is the largest with values in the range 10^-6 ^to 10^-9^, and the Brownian force is the smallest with values in the range 10^-25 ^to 10^-27^. The results are in agreement with the finding of Lazarus et al. [19] that cylindrical-shaped particles lead to a higher thermal transport over spherical-shaped particles. Gao et al. [20] also concluded that the non-spherical nanoparticle shape is helpful in achieving appreciable enhancement of effective thermal conductivity.

### Effect of particle size

**Figure 2:**
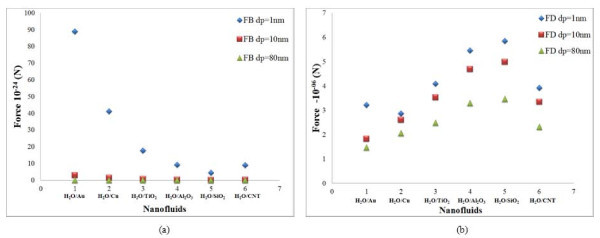
**Effect of particle size in cylindrical particles**. (a) Brownian force and (b) drag force for water-based nanofluids.

**Figure 3:**
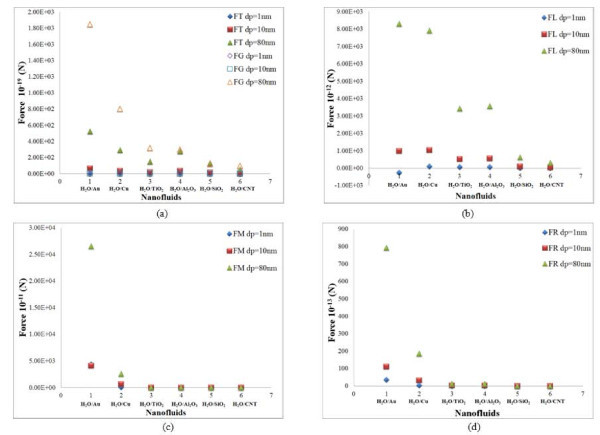
**Effects of particle size in cylindrical particles**. (a) Gravity and thermophoresis forces, (b) lift force, (c) Magnus force, and (d) rotational force for water-based nanofluids.

The results of the scaling analysis on particle size are plotted for cylindrical-shaped nanoparticles in Figures 2 and 3. Here, size of the cylindrical particles is representative of its diameter. From the Figure 2, it is observed that the Brownian force decreases with increasing size of the particles. This is due to the fact that as the particle size increases, the probability for random motion comes down. For a particle size of 1 nm, the Brownian force is 10 orders of magnitude higher compared to that of a particle with size of 80 nm. It is observed that the drag force decreases with increase in size for cylindrical particles. All the other forces show an increase in magnitude with increasing particle diameter.

### Effect of particle concentration

**Figure 4:**
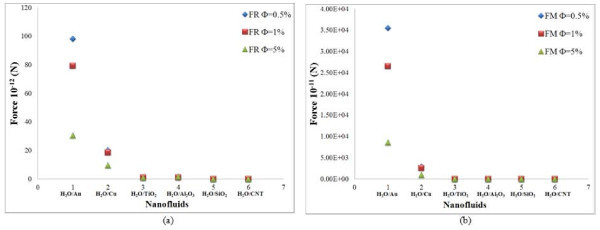
**Effect of particle concentration in cylindrical particles**. (a) Rotational force and (b) Magnus force for water-based nanofluids.

**Figure 5:**
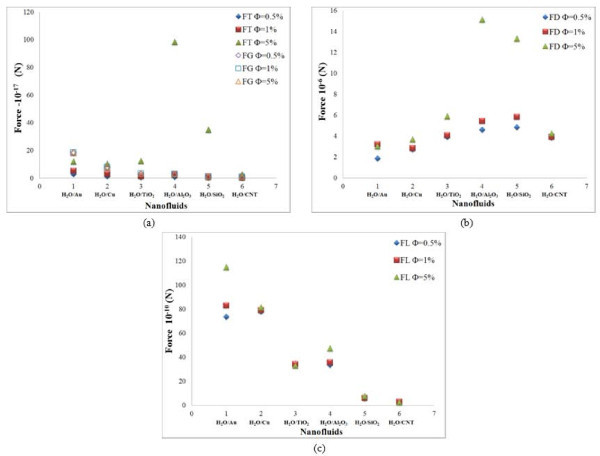
**Effect of particle concentration in cylindrical particles**. (a) Gravity and thermophoresis forces, (b) drag force, and (c) lift force for water-based nanofluids.

**Figure 6:**
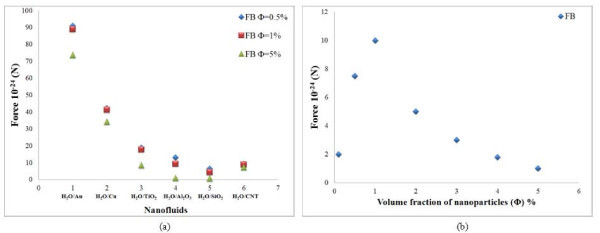
**Effect of particle concentration in cylindrical particles**. (a) Brownian force for water-based nanofluids and (b) Brownian force for alumina-water nanofluid.

The results of the scaling analysis on the particle concentration are plotted for 10-nm cylindrical-shaped particles in Figures 4 and 5. From this figure, it is seen that the rotational and Magnus forces decrease continuously with increasing particle concentration whereas the thermophoresis, drag, and lift forces increase continuously with increasing volume fraction of nanoparticles. It is observed that the variation of gravity force with volume fraction is very small. The effect of volume fraction on Brownian force is also obtained from Figure 6. The Brownian force is initially found to increase with concentration of particles and reaches a maximum value at 1% concentration. Further increase in the concentration reduces the effect of this force.

### Effect of particle temperature

**Figure 7:**
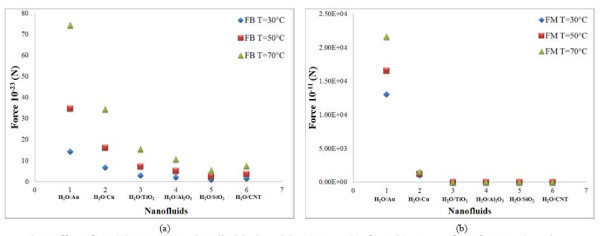
**Effect of particle temperature in cylindrical particles**. (a) Brownian force and (b) Magnus force for water-based nanofluids.

**Figure 8:**
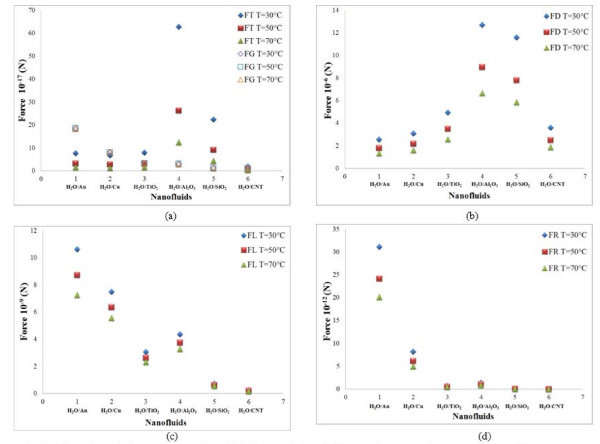
**Effect of particle temperature in cylindrical particles**. (a) Gravity and thermophoresis forces, (b) drag force, (c) lift force, and (d) rotational force for water-based nanofluids.

The results of the scaling analysis on the particle temperature are plotted in Figures 7 and 8. The Brownian and Magnus forces are found to increase with increasing temperature. An increase in the temperature of nanofluids augments the chaotic movement of the suspended nanoparticles thereby increasing the force due to Brownian motion. Gravity force has very little impact with temperature. All other forces including drag, thermophoresis, Saffman's lift, and rotational forces are found to decrease with increasing temperature. This is because for a fixed temperature gradient, the thermophoresis force is inversely proportional to the temperature of the nanofluid as given by Equations 17 and 18.

### Slip mechanisms in different nanofluids

The role of slip mechanisms in different nanofluids is also understood from the scaling analysis in Figures 1, 2, 3, 4, 5, 6, 7, and 8, and it is observed that for gold and copper nanoparticles suspended in water, the values of gravity, Saffman's, Brownian, Magnus, and rotational forces are higher than that of other nanoparticles. The value of drag force is found to be higher in silica- and alumina-based nanofluids. The value of thermophoresis force is also found to be very high in alumina-based nanofluids. The trend observed for water-based nanofluid is found to hold good for ethylene glycol-based nanofluids as well. Comparisons of forces in both the nanofluids are discussed below. The Brownian force is found to be 100 orders of magnitude higher in water-based nanofluids as compared to ethylene glycol-based nanofluids due to higher viscosity of water-based nanofluids. Thermophoresis force is 10 orders of magnitude higher in ethylene glycol-based nanofluids compared to water-based nanofluids. The value of rotational and drag forces are an order of magnitude higher in ethylene glycol-based nanofluids. However, the values of gravity and Magnus forces are found to be an order of magnitude higher in water-based nanofluids.

### Time scale

**Figure 9:**
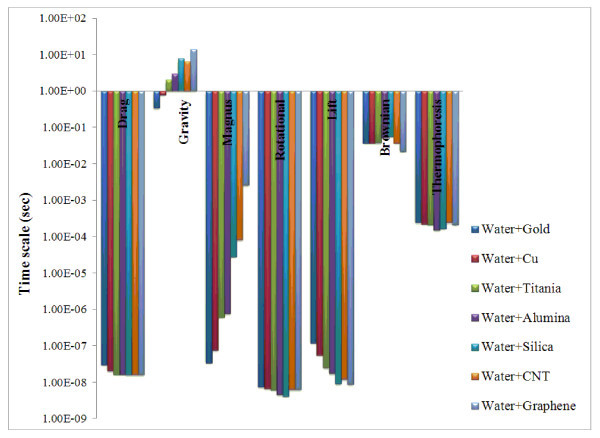
**Time scales of slip mechanisms for water-based nanofluids**.

**Figure 10:**
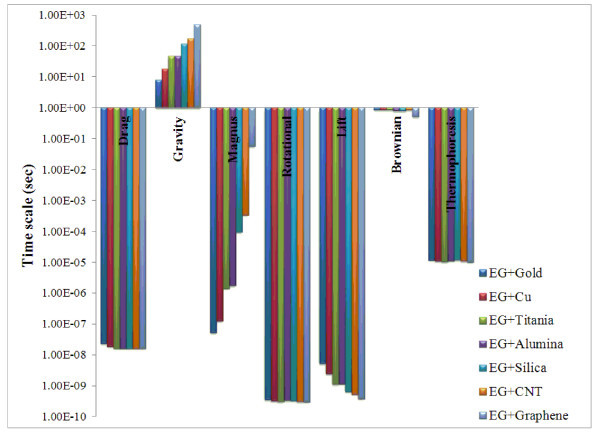
**Time scales of slip mechanisms for ethylene glycol-based nanofluids**.

Time scale is defined as the ratio of the particle size to the velocity of each mechanism (Section 4). Time scale is an important parameter for the comparison of different slip mechanisms in scaling analysis [5]. The results of the time scale analysis for both water- and ethylene glycol-based nanofluids are plotted in Figures 9 and 10. From these figures, it is observed that for water- and ethylene glycol-based nanofluids, the time scales associated with rotational and lift forces are smaller than that of the other forces. It is also found that drag, rotational, and lift forces in both the nanofluids occur within a very short duration of the order of 10^-7 ^to 10^-10 ^s, whereas the time scales of Magnus and thermophoresis forces are in the range between 10^-4 ^and 10^-8 ^s. The time scale of Brownian and gravity forces are in the range of 10^-2 ^to 10^-1 ^and 1 to 100 s, respectively. Hence, the Brownian and gravity tend to act upon for a considerably longer duration than the other forces.

### Role of slip mechanisms on heat transfer augmentation

In this section, the role of slip mechanisms on heat transfer augmentation is studied. The correlation for the turbulent flow of nanofluids inside a tube developed by Xuan and Li [2] is used to calculate the Nusselt number (Nu_nf_), for copper-water nanofluid:(45)

**Figure 11:**
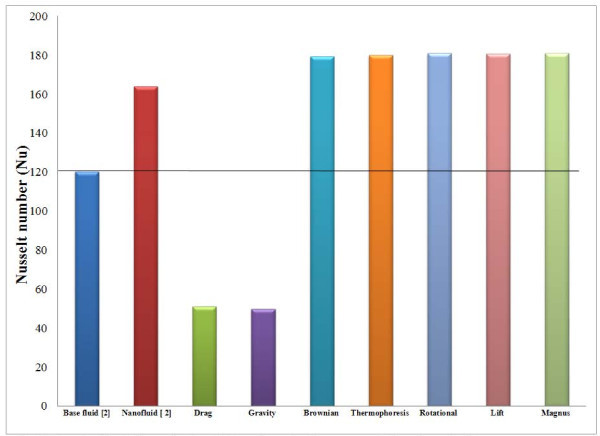
**The effect of slip mechanisms on Nusselt number for copper-water-based nanofluid**.

Here Pe_d _is defined as the particle Peclet number. Particle Peclet number is the product of the particle Reynolds and Prandtl number. The particle Reynolds number in Equation 42 is a function of Reynolds numbers for different slip mechanisms. Thus, it can be inferred that slip mechanisms has an effect on heat transfer augmentation in nanofluids. The Nusselt number of the base fluid corresponding to a pipe diameter of 10 mm is found to be 120. For copper-water nanofluid, the effect of each slip mechanism on the Nusselt number is calculated for a particle concentration of 2%, and diameter of 1 nm is plotted in Figure 11. The heat transfer enhancement in copper-water nanofluid is found to be approximately 36% higher than that of the base fluid comparatively. The effect of each slip mechanisms on heat transfer is discussed below. The drag and gravity forces tend to reduce the Nusselt number of the nanofluid due to their adverse effect on heat transfer, whereas the other forces (Brownian, thermophoresis, lift, rotational, Magnus) contribute to heat transfer augmentation.

## Conclusions

The objective of this work is to understand the effect of nanoparticle slip mechanisms in different nanofluids through scaling analysis. The role of each mechanism is studied by considering the parameters of nanoparticle such as its shape, size, concentration, and temperature. From the scaling analysis, it is found that all of the slip mechanisms are dominant in particles of cylindrical shape as compared to the spherical and sheet particles. The parametric study on the effect of nanoparticle size is conducted by considering the particle size in the range of 1-80 nm. It is found that the magnitudes of slip mechanisms are higher for particles of large size between 10 and 80 nm. However, the Brownian and drag forces are found to dominate in smaller particles below 10 nm. The volume fraction of the nanoparticles in the suspension plays an important role in the thermophysical properties of the nanofluids. The random motion of the particles is enhanced in dilute suspensions; hence, Brownian force dominates at lower volumetric loading of nanoparticles. The Brownian and Magnus forces are found to be dominating at higher temperatures while the other forces are negligible.

With respect to forces, it is found that the Brownian force is more active for nanoparticles of cylindrical shape and in smaller-sized particles with a volumetric concentration of 1%. This force is predominant in water-based nanofluids due to higher viscosity as compared to ethylene glycol-based nanofluids. Thermophoresis force is found to be dominant for cylindrical nanoparticles at lower temperature of nanofluid. The drag force is found to increase with the increasing volume fraction and temperature of the nanoparticles and decrease with the increasing particle size. In terms of time scales, the Brownian and gravity forces act considerably over a longer duration than the other forces. This result holds good for both water- and ethylene glycol-based nanofluids. For copper-water-based nanofluids, the effective contributions of slip mechanisms lead to a heat transfer augmentation which is approximately 36% over that of the base fluid. The drag and gravity forces tend to reduce the Nusselt number of the nanofluid while the other forces tend to enhance it.

### Nomenclature

*A*, actual surface area of the non-spherical particle; *A*, area (m^2^); *A*_sp_, surface area of the sphere of the same volume as the non-spherical particles; *C*, specific heat (J/kg K); *C*_m_, coefficient in Equation 18; *C*_s_, coefficient in Equation 18; *C*_t_, coefficient in Equation 18; *d*_p_, particle diameter (nm); *D*, diameter of the tube (m); *d*_m_, molecular diameter; *F*, force acting on the particle (N); *g*, gravity acceleration (m/s^2^); *h*, length of the cylindrical particle; *I*, unit vector; *k*, thermal conductivity (W/mK); *K*, thermal conductivity ratio (*k*_nf_/*k*_p_); *K*_B_, Boltzmann constant = 1.3806504 × 10^-23 ^(J/K); Kn, Knudsen number; *K*_L_, coefficient in Equation 19; *m*_p_, mass of the particle (kg); , mass flow rate of the particle (kg/s); *N*_A_, Avagadro's number = 6.0221367 × 10^23^/mol; Nu, Nusselt number; *P*, pressure (Pa); Pe, Peclet number; *R*, radius of the tube (m); *R*, universal gas constant = 8.3145 J/mol.K; *Re*, Reynolds number; *S*_p_, source term; *t*, time (s); *T*, temperature (K); *T*_bf_, stress tensor of nanofluids; *v*, velocity (m/s); *V*, relative velocity of nanofluid (m/s); *V*_p_, volume of the particle (m^3^); *z*, location; *z*, location inside the tube (m)

### Greeks symbols

*β*, inter-phase momentum exchange coefficient; , shear rate (s^-1^); *λ*, mean free path of the fluid (m); *μ*, dynamic viscosity (kg/ms); Ψ, shape factor; *ρ*, fluid density (kg/m^3^); *τ*, time (s); *τ*_p_, relaxation time of the particle; *ϕ*, volume fraction of the nanoparticle; *ν*, kinematic viscosity (m^2^/s)

### Subscripts

B, Brownian; bf, base fluids; D, drag; G, gravity; L, lift; m, mean; M, Magnus; nf, nanofluids; p, particle; R, rotational; T, thermophoresis

## Competing interests

The authors declare that they have no competing interests.

## Authors' contributions

SS carried out the scaling analysis for each slip mechanisms and also the effect of each slip mechanisms in heat transfer enhancement. AP drafted the manuscript, together with contributions to the analysis of the results and in discussions on the technical content. SKD participated in the finalization of manuscript and in discussions on the technical content. All authors read and approved the final manuscript.
